# *S*-Benzyl 3-[1-(6-methyl­pyridin-2-yl)ethyl­idene]di­thio­carbazate: crystal structure and Hirshfeld surface analysis

**DOI:** 10.1107/S2056989018001330

**Published:** 2018-01-31

**Authors:** Siti Aminah Omar, Chee Keong Chah, Thahira B. S. A. Ravoof, Mukesh M. Jotani, Edward R. T. Tiekink

**Affiliations:** a Department of Chemistry, Faculty of Science, Universiti Putra Malaysia, 43400 UPM Serdang, Selangor Darul Ehsan, Malaysia; b Department of Physics, Bhavan’s Sheth R. A. College of Science, Ahmedabad, Gujarat 380 001, India; c Research Centre for Crystalline Materials, School of Science and Technology, Sunway University, 47500 Bandar Sunway, Selangor Darul Ehsan, Malaysia

**Keywords:** crystal structure, di­thio­carbazate ester, hydrogen bonding, Hirshfeld surface analysis

## Abstract

In the title di­thio­carbazate ester, C_16_H_17_N_3_S_2_ (systematic name: (*Z*)-{[(benzyl­sulfan­yl)methane­thio­yl]amino}[1-(6-methyl­pyridin-2-yl)ethyl­idene]amine), the central methyl­idenehydrazinecarbodi­thio­ate (C_2_N_2_S_2_) core is almost planar (r.m.s. deviation = 0.0111 Å) and forms dihedral angles of 71.67 (3)° with the approximately orthogonally inclined thio­ester phenyl ring, and 7.16 (7)° with the approximately coplanar substituted pyridyl ring. The latter arrangement and the *Z* configuration about the imine-C=N bond allows for the formation of an intra­molecular hydrazine-N—H⋯N(pyrid­yl) hydrogen bond that closes an *S*(6) loop. In the crystal, phenyl-C—H⋯S(thione), methyl­ene-C—H⋯π(pyrid­yl), methyl­ene- and phenyl-C—H⋯π(phen­yl) contacts connect mol­ecules into supra­molecular layers propagating in the *bc* plane; the layers stack along the *a* axis with no directional inter­actions between them. The analysis of the Hirshfeld surface indicates the relative importance of an intra­layer phenyl-H⋯H(pyrid­yl) contact upon the mol­ecular packing.

## Abstract 2

The title mol­ecule has a approximately coplanar relationship between the methyl­idenehydrazinecarbodi­thio­ate (C_2_N_2_S_2_) core and substituted pyridyl ring but the former plane is nearly orthogonal to the thio­ester phenyl ring. Supra­molecular layers in the *bc* plane sustained by C—H⋯S and C—H⋯π inter­actions feature in the crystal.

## Chemical context   

Di­thio­carbaza­tes are compounds that contain both nitro­gen and sulfur donor atoms, which can react with ketones or aldehydes, *via* condensation, to yield Schiff bases. Different ligands can be obtained by introducing different organic substituents, which causes variation in their biological properties, although they may differ only slightly in their mol­ecular structures (Ali *et al.*, 1977 ▸; Tarafder *et al.*, 2001 ▸, 2002 ▸). Inter­est in this class of compound remains high as studies have shown that they possess anti-cancer (Mirza *et al.*, 2014 ▸), anti-bacterial (Bhat *et al.*, 2018 ▸), anti-fungal (Nithya *et al.*, 2017 ▸), anti-viral (Chew *et al.*, 2004 ▸) and anti-inflammatory (Zangrando *et al.*, 2015 ▸) properties. Pyridine derivatives have also been a subject of much inter­est since the 1930′s with the discovery of niacin for the treatment of dermatitis and dementia (Henry, 2004 ▸). 3-Amino­pyridine­carbaldehyde thio­semicarbazone is another pyridine-containing compound that has shown promising activity in advanced leukemia patients in a clinical phase I evaluation (Karp *et al.*, 2008 ▸). Although considerable work has been conducted on pyridine-derived Schiff bases and their biological activities, we report, as part of our research into the synthesis and characterization of pyridine-based Schiff bases and their metal complexes, the crystal structure and Hirshfeld surface analysis of a potentially tridentate Schiff base derived from the condensation of *S*-benzyl­dithio­carbazate with 2-acetyl-6-methyl pyridine.
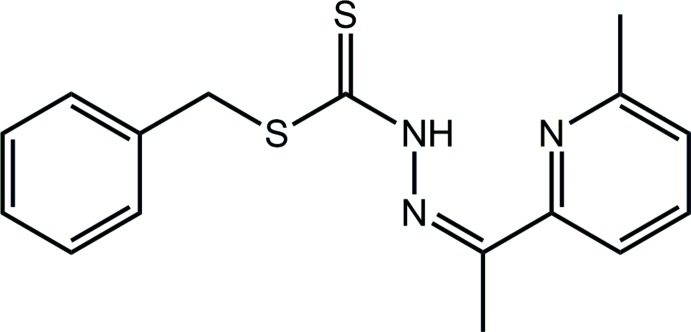


## Structural commentary   

**Figure 1:**
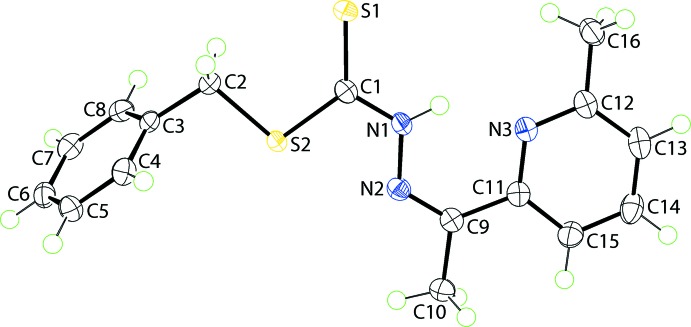
The mol­ecular structure of (I), showing the atom-labelling scheme and displacement ellipsoids at the 70% probability level.

The mol­ecular structure of (I), Fig. 1 ▸, comprises three distinct almost planar residues with the central methyl­idenehydrazinecarbodi­thio­ate, C_2_N_2_S_2_, chromophore [r.m.s. deviation = 0.0111 Å] being flanked by the thio­ester-phenyl ring and the substituted pyridyl ring, forming dihedral angles of 71.67 (3) and 7.16 (7)°, respectively, indicating nearly orthogonal and co-planar dispositions, respectively; the dihedral angle between the outer rings is 65.79 (4)°. The configuration about the imine-C9=N2 bond [1.2924 (18) Å] is *Z*, resulting in the hydrazine-N1—H hydrogen atom being directed towards the pyridyl-N3 atom, enabling the formation of an inter­molecular amine-N1—H⋯N3(pyrid­yl) hydrogen bond that closes an *S*(6) loop, Table 1 ▸. The pyridyl-methyl group is *syn* with the thione-S1 atom and at the same time is orientated to the opposite side of the mol­ecule to the imine-bound methyl group.

## Supra­molecular features   

**Figure 2:**
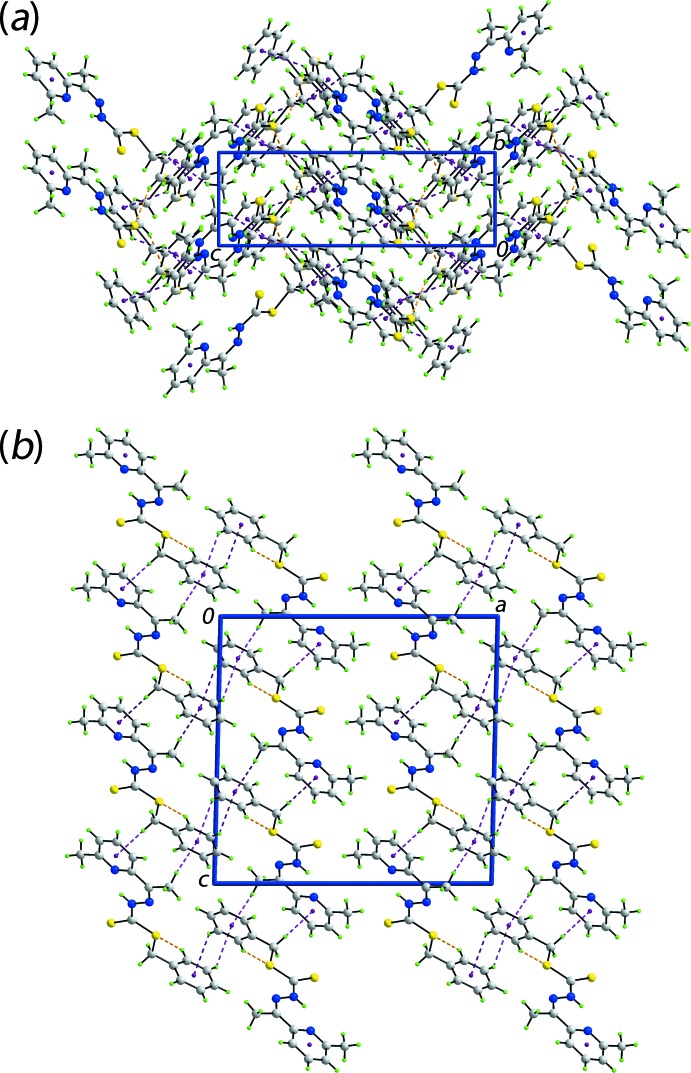
Mol­ecular packing in (I), showing (*a*) a view of the supra­molecular layer sustained by C—H⋯S and C—H⋯π inter­actions and (*b*) a view of the unit-cell contents shown in projection down the *b* axis, highlighting the stacking of layers. The C—H⋯S and C—H⋯π inter­actions are shown as orange and purple dashed lines, respectively.

The participation of the hydrazine-N1—H hydrogen and pyridyl-N3 atoms in the intra­molecular N—H⋯N hydrogen bond precludes their participation in inter­molecular inter­actions. The mol­ecular packing features weak phenyl-C8—H⋯S2(thione) inter­actions, leading to chains along the *b*-axis direction, and a number of C—H⋯π contacts, *i.e*. methyl­ene-C2—H⋯π(pyrid­yl), phenyl-C7—H⋯π(phen­yl) and methyl-C10—H⋯π(phen­yl), as detailed in Table 1 ▸. The aforementioned contacts link mol­ecules into supra­molecular layers in the *bc* plane, Fig. 2 ▸ *a*. Layers stack along the *a* axis with no directional inter­actions between them, Fig. 2 ▸ *b*.

## Analysis of the Hirshfeld surfaces   

**Figure 3:**
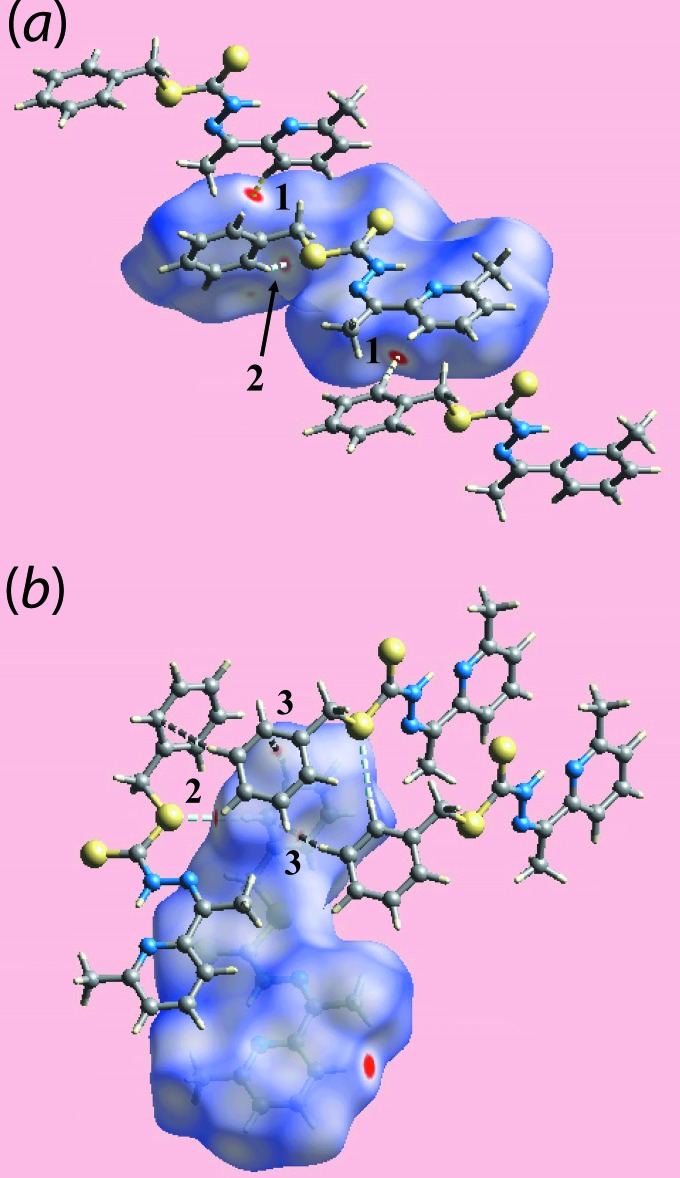
Views of Hirshfeld surface mapped over *d*_norm_ for (I): (*a*) in the range −0.120 to +1.541 au highlighting short inter­atomic H⋯H contacts with yellow dashed lines and label ‘1’ and (*b*) in the range −0.050 to +1.541 au highlighting short inter­atomic C⋯H/H⋯C contacts with black dashed lines and label ‘3’. Weak inter­molecular C—H⋯S/S⋯H—C contacts are indicated by sky-blue dashed lines and label ‘2’ in both (*a*) and (*b*).

**Figure 4:**
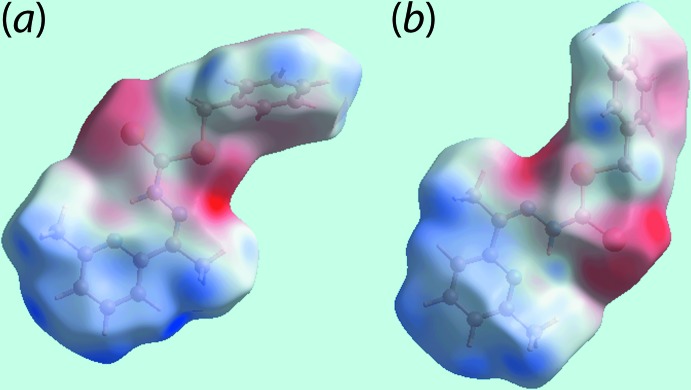
Two views of the Hirshfeld surface mapped over the electrostatic potential for (I) in the range ±0.055 au. The red and blue regions represent negative and positive electrostatic potentials, respectively.

**Figure 5:**
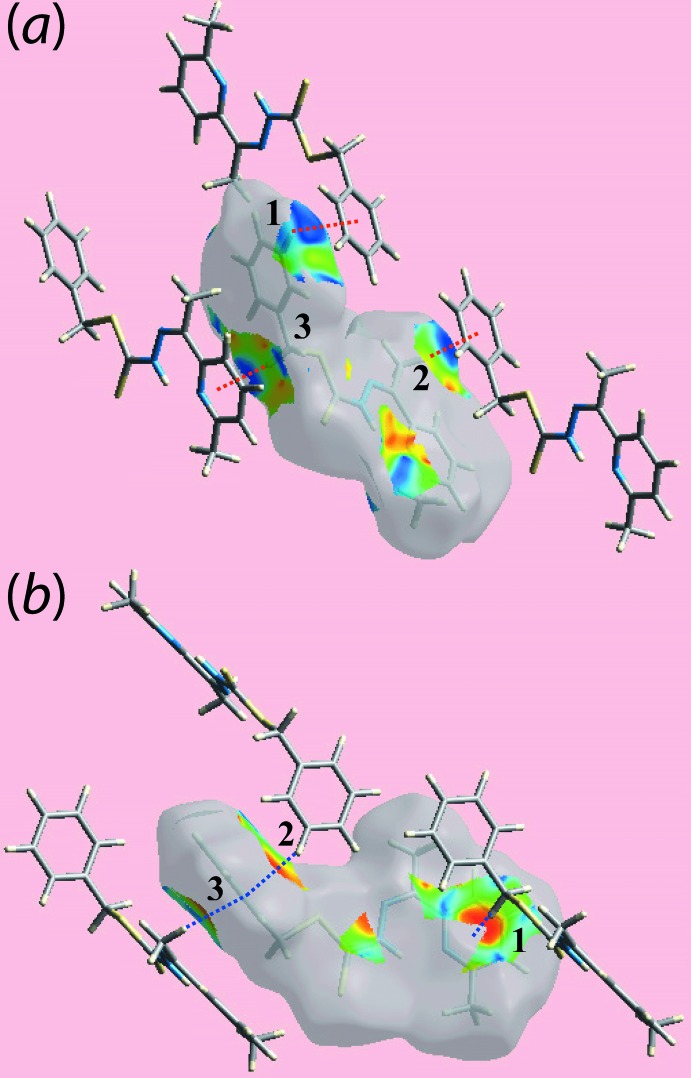
Two views of Hirshfeld surface mapped with shape-index properties for (I) highlighting (*a*) C—H⋯π contacts and (*b*) their reciprocal *i.e*. π⋯H—C contacts, with red and blue dotted lines, respectively, and labels ‘1’–‘3’.

The Hirshfeld surfaces calculated for (I) were performed in accord with recent studies on an organic mol­ecule (Tan *et al.*, 2017 ▸) and serve to provide insight into the influence of different inter­molecular inter­actions in the crystal. A very short (2.23 Å) intra-layer H⋯H contact between the phenyl-H4 and pyridyl-H15 atoms (Table 2 ▸) is significant in the crystal of (I) and is viewed as the bright-red spots near these atoms on the Hirshfeld surface mapped over *d*_norm_ in Fig. 3 ▸ *a* (labelled as ‘1’). The presence of the weak inter­molecular C—H⋯S contact involving the phenyl-C8 and thione-S2 atoms is evident from the diminutive red spots near these atoms in Fig. 3 ▸ (labelled as ‘2’). The faint-red spots near the phenyl-H7 and -C8 atoms in Fig. 3 ▸ *b* (labelled as ‘3’) characterize the short surface C⋯H/H⋯C contacts and indicate the relative importance of this particular C—H⋯π contact compared with the other two C—H⋯π contacts summarized in Table 1 ▸. The most prominent inter­layer contact appears to be a weak methyl-C16—H⋯S1(ester) inter­action (Table 2 ▸). The donors and acceptors of inter­molecular inter­actions are also represented with blue and red regions, respectively, corresponding to positive and negative electrostatic potentials on the Hirshfeld surface mapped over electrostatic potential in Fig. 4 ▸. The inter­molecular C—H⋯π contacts, involving donor atoms, and their reciprocal contacts, *i.e*. π⋯H—C, containing π-bond acceptors, on the Hirshfeld surface mapped with the shape-index property are illustrated in Fig. 5 ▸.

**Figure 6:**
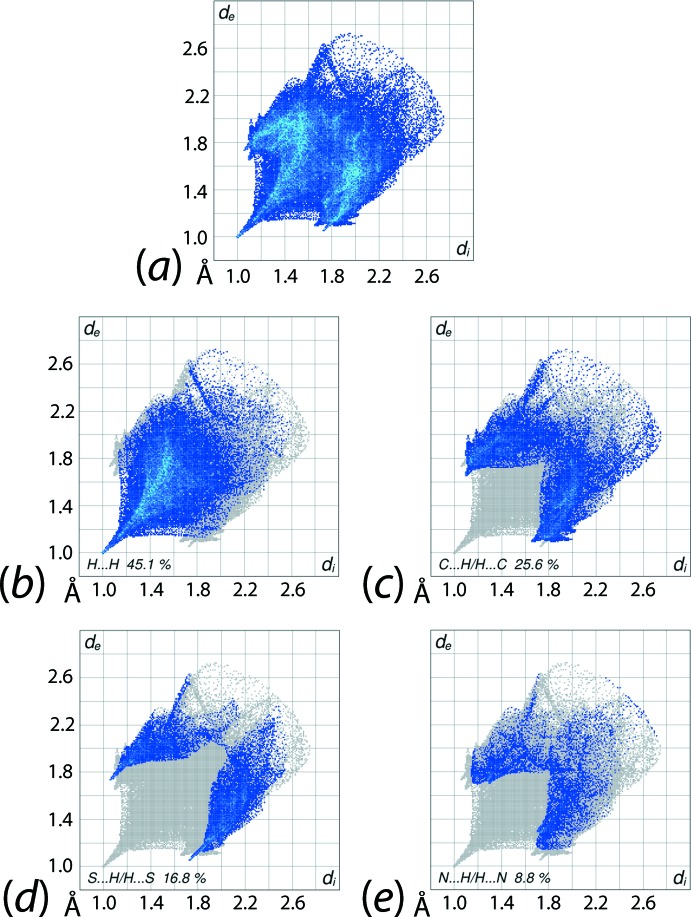
(*a*) The full two-dimensional fingerprint plot for (I) and fingerprint plots delineated into (*b*) H⋯H, (*c*) C⋯H/H⋯C, (*d*) S⋯H/H⋯S and (*e*) S⋯H/H⋯S contacts.

The overall two-dimensional fingerprint plot for (I), Fig. 6 ▸ *a*, and those delineated (McKinnon *et al.*, 2007 ▸) into H⋯H, C⋯H/H⋯C, S⋯H/H⋯S and N⋯H/H⋯N contacts are illustrated in Fig. 6 ▸ *b*–*e*; the percentage contributions from the different inter­atomic contacts to the Hirshfeld surface are summarized in Table 3 ▸. The single tip at *d*_e_ + *d*_i_ ∼ 2.0 Å near the vertex of the cone-shaped distribution of points in the fingerprint plot delineated into H⋯H contacts (Fig. 6 ▸ *b*) indicate the significant influence of the short inter­atomic phenyl-H⋯H(pyrid­yl) contacts in the crystal mentioned above. The inter­molecular C—H⋯π inter­actions discussed earlier are characterized by short inter­atomic C⋯H/H⋯C contacts (Table 2 ▸) and their presence are indicated by the distribution of points around a pair of peaks at *d*_e_ + *d*_i_ ∼ 2.8 Å in Fig. 6 ▸ *c*, and by the concave surfaces around the phenyl (C3–C8) and pyridyl (N3,C11–C15) rings on the Hirshfeld surface mapped over the electrostatic potential in Fig. 4 ▸. The inter­molecular C8—H8⋯S2 contact in the crystal is characterized by the pair of forceps-like tips at *d*_e_ + *d*_i_ ∼ 2.8 Å in Fig. 6 ▸ *d*. The inter­atomic N⋯H/H⋯N contacts do not represent directional inter­actions as the inter­atomic separations are greater than sum of their van der Waals radii as evident from Fig. 6 ▸ *e*. Similarly, the other surface contacts summarized in Table 3 ▸ have negligible effect on the packing.

## Database survey   

As mentioned in the *Chemical context*, there is sustained inter­est in this class of compound and this is reflected by the observation there are four closely related structures available for comparison, varying in the *S*-bound group and substitution in the 2-pyridyl ring. In common with (I), the derivative with the 4-methyl­benzyl ester and with a methyl group in the 5-position of the pyridyl ring, a *Z*-configuration is noted about the imine bond allowing for the formation of an intra­molecular hydrazine-N—H⋯N(pyrid­yl) hydrogen bond (Ravoof *et al.*, 2015 ▸). By contrast, the three remaining analogues, *i.e*. the methyl ester with no substitution in the pyridyl ring (Basha *et al.*, 2012 ▸), benzyl ester/4-methyl­pyridyl and 4-methyl­benzyl ester/4-methyl­pyridyl (Omar *et al.*, 2014 ▸), an *E*-configuration is found about the imine bond, a disposition that allows for the formation of inter­molecular thio­amide-N—H⋯S(thione) hydrogen bonds and eight-membered {⋯HNCS}_2_ synthons.

## Synthesis and crystallization   

All chemicals were of analytical grade and were used without any further purification. *S*-Benzyl­dithio­carbazate (SBDTC) was prepared according to the method published by Ali & Tarafder (1977 ▸). Potassium hydroxide (11.4 g, 0.2 mol) was dissolved in absolute ethanol (70 ml) and to this solution hydrazine hydrate (10 g, 0.2 mol) was added. The mixture was then cooled in an ice bath followed by the dropwise addition of carbon di­sulfide (15.2 g, 0.2 mol) with constant stirring over 1 h. The two layers that formed were then separated using a separating funnel. The brown organic lower layer was dissolved in 40% ethanol. Benzyl chloride (25 ml, 0.2 mol) was then added dropwise into the mixture with vigorous stirring. The white product that formed was filtered off, washed with cold ethanol and dried in a desiccator over anhydrous silica gel. Pure SBDTC was obtained by recrystallization using absolute ethanol as the solvent. Yield: 75%, m.p. 397–399 K. SBDTC (1.98 g, 0.01 mol) was subsequently dissolved in hot aceto­nitrile (100 ml) and added to an equimolar solution of 2-acetyl-6-methyl pyridine (1.35 g, 0.01 mol) in ethanol (25 ml). The mixture was then heated on a water bath until the volume reduced to half. A yellow precipitate formed upon standing at room temperature for 1 h which was washed with cold ethanol. A small amount of product was dissolved in aceto­nitrile and left to stand for a week, after which yellow prisms suitable for single-crystal X-ray diffraction analysis formed. IR (cm^−1^): 2921 ν(N—H), 1560 ν(C=N), 1055 ν(N—N), 881 ν(CSS).

## Refinement   

Crystal data, data collection and structure refinement details are summarized in Table 4 ▸. The carbon-bound H atoms were placed in calculated positions (C—H = 0.95–0.99 Å) and were included in the refinement in the riding-model approximation, with *U*_iso_(H) set to 1.2–1.5*U*_eq_(C). The nitro­gen-bound H atom was located in a difference Fourier map, but was refined with a distance restraint of N—H = 0.88±0.01 Å, and with *U*_iso_(H) set to 1.2*U*_eq_(N).

## Supplementary Material

Crystal structure: contains datablock(s) I, global. DOI: 10.1107/S2056989018001330/hb7730sup1.cif

Structure factors: contains datablock(s) I. DOI: 10.1107/S2056989018001330/hb7730Isup2.hkl

Supporting information file. DOI: 10.1107/S2056989018001330/hb7730Isup3.cml

CCDC reference: 1818384

Additional supporting information:  crystallographic information; 3D view; checkCIF report

## Figures and Tables

**Table 1:** Hydrogen-bond geometry (Å, °) *Cg*1 and *Cg*2 are the centroids of the (N3,C11–C15) and (C3—C8) rings, respectively.

*D*—H⋯*A*	*D*—H	H⋯*A*	*D*⋯*A*	*D*—H⋯*A*
N1—H1*N*⋯N3	0.87 (1)	1.91 (2)	2.6123 (16)	138 (1)
C8—H8⋯S2^i^	0.95	2.90	3.6184 (16)	154
C2—H2*A*⋯*Cg*1^ii^	0.99	2.76	3.5325 (15)	135
C7—H7⋯*Cg*2^iii^	0.95	2.89	3.5760 (16)	130
C10—H10*C*⋯*Cg*2^iv^	0.98	2.68	3.5842 (15)	153

Symmetry codes: (i) 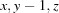; (ii) 
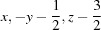
; (iii) 
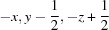
; (iv) 
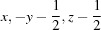
.

**Table 2:** Summary of short surface contacts (Å) in (I)

Contact	Distance	Symmetry operation
H4⋯H15	1.98	*x*, 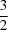 − *y*, − 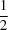 + *z*
H7⋯C8	2.74	-*x*, − 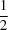 + *y*, 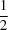 − *z*
H2*A*⋯C11	2.85	*x*, 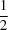 − *y*, − 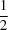 + *z*
H10*C*⋯ C3	2.86	*x*, 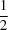 − *y*, 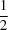 + *z*
H16*C*⋯S1	3.05	-*x*, −*y*, −*z*

**Table 3:** Relative percentage contributions of close contacts to the Hirshfeld surface of (I)

H⋯H	45.1
C⋯H/H⋯C	25.6
S⋯H/H⋯S	16.8
N⋯H/H⋯N	8.8
C⋯S/S⋯C	2.1
S⋯N/N⋯S	0.9
C⋯C	0.7

**Table 4:** Experimental details

Crystal data	
Chemical formula	C_16_H_17_N_3_S_2_
*M*_r_	315.44
Crystal system, space group	Monoclinic, *P*2_1_/*c*
Temperature (K)	100
*a*, *b*, *c* (Å)	17.0395 (3), 5.5354 (1), 16.4292 (2)
β (°)	91.355 (1)
*V* (Å^3^)	1549.18 (4)
*Z*	4
Radiation type	Cu *K*α
μ (mm^−1^)	3.08
Crystal size (mm)	0.28 × 0.18 × 0.08

Data collection	
Diffractometer	Rigaku Oxford Diffraction Gemini E
Absorption correction	Multi-scan (*CrysAlis PRO*; Agilent, 2011 ▸)
*T*_min_, *T*_max_	0.561, 1.000
No. of measured, independent and observed [*I* > 2σ(*I*)] reflections	29598, 3000, 2888
*R*_int_	0.034

Refinement	
*R*[*F* ^2^ > 2σ(*F* ^2^)], *wR*(*F* ^2^), *S*	0.032, 0.092, 1.04
No. of reflections	3000
No. of parameters	195
No. of restraints	1
H-atom treatment	H atoms treated by a mixture of independent and constrained refinement
Δρ_max_, Δρ_min_ (e Å^−3^)	0.40, −0.26

Computer programs: *CrysAlis PRO* (Agilent, 2011 ▸), *SHELXS97* (Sheldrick, 2008 ▸), *SHELXL2014* (Sheldrick, 2015 ▸), *ORTEP-3 for Windows* (Farrugia, 2012 ▸), *DIAMOND* (Brandenburg, 2006 ▸) and *publCIF* (Westrip, 2010 ▸).

## supplementary crystallographic information

### Crystal data

**Table:**

C_16_H_17_N_3_S_2_	*F*(000) = 664
*M**_r_* = 315.44	*D*_x_ = 1.352 Mg m^−^^3^
Monoclinic, *P*2_1_/*c*	Cu *K*α radiation, λ = 1.5418 Å
*a* = 17.0395 (3) Å	Cell parameters from 16086 reflections
*b* = 5.5354 (1) Å	θ = 3.7–71.4°
*c* = 16.4292 (2) Å	µ = 3.08 mm^−^^1^
β = 91.355 (1)°	*T* = 100 K
*V* = 1549.18 (4) Å^3^	Prism, yellow
*Z* = 4	0.28 × 0.18 × 0.08 mm

### Data collection

**Table:**

Rigaku Oxford Diffraction Gemini E diffractometer	3000 independent reflections
Radiation source: Enhance X-ray source	2888 reflections with *I* > 2σ(*I*)
Mirror monochromator	*R*_int_ = 0.034
Detector resolution: 16.1952 pixels mm^-1^	θ_max_ = 71.5°, θ_min_ = 5.2°
ω scan	*h* = −20→20
Absorption correction: multi-scan (CrysAlis PRO; Agilent, 2011)	*k* = −6→6
*T*_min_ = 0.561, *T*_max_ = 1.000	*l* = −20→20
29598 measured reflections	

### Refinement

**Table:**

Refinement on *F*^2^	1 restraint
Least-squares matrix: full	Hydrogen site location: mixed
*R*[*F*^2^ > 2σ(*F*^2^)] = 0.032	H atoms treated by a mixture of independent and constrained refinement
*wR*(*F*^2^) = 0.092	*w* = 1/[σ^2^(*F*_o_^2^) + (0.0635*P*)^2^ + 0.6508*P*] where *P* = (*F*_o_^2^ + 2*F*_c_^2^)/3
*S* = 1.04	(Δ/σ)_max_ < 0.001
3000 reflections	Δρ_max_ = 0.40 e Å^−^^3^
195 parameters	Δρ_min_ = −0.26 e Å^−^^3^

### Special details

**Table:**

Geometry. All esds (except the esd in the dihedral angle between two l.s. planes) are estimated using the full covariance matrix. The cell esds are taken into account individually in the estimation of esds in distances, angles and torsion angles; correlations between esds in cell parameters are only used when they are defined by crystal symmetry. An approximate (isotropic) treatment of cell esds is used for estimating esds involving l.s. planes.

### Fractional atomic coordinates and isotropic or equivalent isotropic displacement parameters (Å^2^)

**Table:**

	*x*	*y*	*z*	*U*_iso_*/*U*_eq_	
S1	0.36900 (2)	0.02321 (6)	0.35206 (2)	0.01816 (12)	
S2	0.20666 (2)	0.21559 (6)	0.30590 (2)	0.01465 (12)	
N1	0.29576 (7)	0.3795 (2)	0.42370 (7)	0.0161 (3)	
H1N	0.3346 (8)	0.396 (3)	0.4582 (9)	0.019*	
N2	0.23272 (7)	0.5327 (2)	0.43063 (7)	0.0156 (3)	
N3	0.35999 (7)	0.5890 (2)	0.55123 (7)	0.0151 (2)	
C1	0.29438 (8)	0.2120 (2)	0.36453 (8)	0.0144 (3)	
C2	0.21915 (8)	−0.0396 (3)	0.23758 (8)	0.0168 (3)	
H2A	0.2609	−0.0060	0.1982	0.020*	
H2B	0.2333	−0.1872	0.2686	0.020*	
C3	0.14062 (8)	−0.0702 (3)	0.19410 (8)	0.0141 (3)	
C4	0.11330 (8)	0.1021 (3)	0.13811 (8)	0.0166 (3)	
H4	0.1456	0.2360	0.1253	0.020*	
C5	0.03983 (8)	0.0805 (3)	0.10104 (8)	0.0182 (3)	
H5	0.0221	0.1992	0.0631	0.022*	
C6	−0.00811 (8)	−0.1147 (3)	0.11931 (8)	0.0178 (3)	
H6	−0.0586	−0.1298	0.0940	0.021*	
C7	0.01836 (9)	−0.2871 (3)	0.17477 (9)	0.0187 (3)	
H7	−0.0140	−0.4211	0.1873	0.022*	
C8	0.09226 (9)	−0.2646 (3)	0.21221 (9)	0.0166 (3)	
H8	0.1097	−0.3829	0.2504	0.020*	
C9	0.23507 (8)	0.6991 (2)	0.48580 (8)	0.0147 (3)	
C10	0.16295 (8)	0.8567 (3)	0.48703 (8)	0.0175 (3)	
H10A	0.1261	0.8058	0.4437	0.026*	
H10B	0.1780	1.0254	0.4783	0.026*	
H10C	0.1379	0.8411	0.5399	0.026*	
C11	0.29926 (8)	0.7469 (3)	0.54680 (8)	0.0146 (3)	
C12	0.41782 (8)	0.6242 (3)	0.60655 (8)	0.0167 (3)	
C13	0.41666 (9)	0.8185 (3)	0.66067 (9)	0.0203 (3)	
H13	0.4580	0.8395	0.6999	0.024*	
C14	0.35505 (9)	0.9797 (3)	0.65674 (9)	0.0215 (3)	
H14	0.3533	1.1126	0.6932	0.026*	
C15	0.29548 (8)	0.9452 (3)	0.59868 (9)	0.0192 (3)	
H15	0.2528	1.0553	0.5945	0.023*	
C16	0.48418 (9)	0.4453 (3)	0.60873 (10)	0.0222 (3)	
H16A	0.4773	0.3303	0.5638	0.033*	
H16B	0.4844	0.3579	0.6606	0.033*	
H16C	0.5341	0.5309	0.6032	0.033*	

### Atomic displacement parameters (Å^2^)

**Table:**

	*U*^11^	*U*^22^	*U*^33^	*U*^12^	*U*^13^	*U*^23^
S1	0.0127 (2)	0.0190 (2)	0.0227 (2)	0.00341 (12)	−0.00205 (14)	−0.00368 (13)
S2	0.01277 (19)	0.0172 (2)	0.01385 (19)	0.00289 (12)	−0.00273 (13)	−0.00297 (12)
N1	0.0123 (6)	0.0196 (6)	0.0162 (6)	0.0017 (5)	−0.0029 (4)	−0.0036 (5)
N2	0.0133 (6)	0.0174 (6)	0.0160 (6)	0.0005 (5)	0.0007 (4)	0.0005 (4)
N3	0.0150 (6)	0.0160 (6)	0.0141 (5)	−0.0020 (5)	−0.0001 (4)	0.0015 (4)
C1	0.0127 (7)	0.0164 (7)	0.0141 (6)	−0.0014 (5)	0.0004 (5)	0.0019 (5)
C2	0.0162 (7)	0.0172 (7)	0.0168 (6)	0.0032 (5)	−0.0014 (5)	−0.0054 (5)
C3	0.0140 (6)	0.0156 (6)	0.0125 (6)	0.0017 (5)	0.0005 (5)	−0.0042 (5)
C4	0.0175 (7)	0.0159 (7)	0.0165 (6)	−0.0040 (5)	0.0016 (5)	0.0003 (5)
C5	0.0197 (7)	0.0201 (7)	0.0146 (6)	0.0014 (6)	−0.0022 (5)	0.0012 (5)
C6	0.0140 (7)	0.0225 (7)	0.0168 (6)	−0.0006 (5)	−0.0001 (5)	−0.0053 (6)
C7	0.0184 (7)	0.0173 (7)	0.0206 (7)	−0.0036 (5)	0.0050 (6)	−0.0025 (5)
C8	0.0194 (7)	0.0150 (7)	0.0155 (6)	0.0025 (5)	0.0025 (5)	0.0004 (5)
C9	0.0140 (7)	0.0163 (7)	0.0137 (6)	−0.0017 (5)	0.0018 (5)	0.0020 (5)
C10	0.0161 (7)	0.0183 (7)	0.0181 (6)	0.0019 (6)	0.0009 (5)	0.0006 (5)
C11	0.0138 (7)	0.0170 (7)	0.0133 (6)	−0.0027 (5)	0.0031 (5)	0.0011 (5)
C12	0.0157 (7)	0.0199 (7)	0.0144 (6)	−0.0046 (5)	0.0000 (5)	0.0036 (5)
C13	0.0185 (7)	0.0269 (8)	0.0155 (7)	−0.0069 (6)	−0.0005 (5)	−0.0009 (6)
C14	0.0209 (8)	0.0251 (8)	0.0188 (7)	−0.0051 (6)	0.0039 (6)	−0.0074 (6)
C15	0.0160 (7)	0.0214 (7)	0.0203 (7)	−0.0004 (6)	0.0036 (5)	−0.0036 (6)
C16	0.0189 (7)	0.0225 (8)	0.0250 (7)	−0.0004 (6)	−0.0064 (6)	0.0010 (6)

### Geometric parameters (Å, º)

**Table:**

S1—C1	1.6624 (14)	C7—C8	1.394 (2)
S2—C1	1.7591 (14)	C7—H7	0.9500
S2—C2	1.8199 (14)	C8—H8	0.9500
N1—C1	1.3432 (18)	C9—C11	1.4895 (19)
N1—N2	1.3754 (16)	C9—C10	1.5074 (19)
N1—H1N	0.865 (9)	C10—H10A	0.9800
N2—C9	1.2924 (18)	C10—H10B	0.9800
N3—C12	1.3390 (18)	C10—H10C	0.9800
N3—C11	1.3554 (18)	C11—C15	1.392 (2)
C2—C3	1.5113 (19)	C12—C13	1.396 (2)
C2—H2A	0.9900	C12—C16	1.503 (2)
C2—H2B	0.9900	C13—C14	1.378 (2)
C3—C8	1.392 (2)	C13—H13	0.9500
C3—C4	1.397 (2)	C14—C15	1.389 (2)
C4—C5	1.384 (2)	C14—H14	0.9500
C4—H4	0.9500	C15—H15	0.9500
C5—C6	1.392 (2)	C16—H16A	0.9800
C5—H5	0.9500	C16—H16B	0.9800
C6—C7	1.387 (2)	C16—H16C	0.9800
C6—H6	0.9500		
			
C1—S2—C2	102.63 (7)	C7—C8—H8	119.7
C1—N1—N2	119.01 (11)	N2—C9—C11	127.48 (13)
C1—N1—H1N	123.2 (12)	N2—C9—C10	114.20 (12)
N2—N1—H1N	117.8 (12)	C11—C9—C10	118.31 (12)
C9—N2—N1	119.09 (12)	C9—C10—H10A	109.5
C12—N3—C11	119.42 (12)	C9—C10—H10B	109.5
N1—C1—S1	121.52 (10)	H10A—C10—H10B	109.5
N1—C1—S2	112.88 (10)	C9—C10—H10C	109.5
S1—C1—S2	125.60 (8)	H10A—C10—H10C	109.5
C3—C2—S2	105.26 (9)	H10B—C10—H10C	109.5
C3—C2—H2A	110.7	N3—C11—C15	121.47 (13)
S2—C2—H2A	110.7	N3—C11—C9	117.98 (12)
C3—C2—H2B	110.7	C15—C11—C9	120.54 (13)
S2—C2—H2B	110.7	N3—C12—C13	121.55 (14)
H2A—C2—H2B	108.8	N3—C12—C16	117.48 (13)
C8—C3—C4	118.60 (13)	C13—C12—C16	120.97 (13)
C8—C3—C2	120.51 (13)	C14—C13—C12	119.43 (13)
C4—C3—C2	120.81 (12)	C14—C13—H13	120.3
C5—C4—C3	120.98 (13)	C12—C13—H13	120.3
C5—C4—H4	119.5	C13—C14—C15	119.11 (14)
C3—C4—H4	119.5	C13—C14—H14	120.4
C4—C5—C6	120.09 (13)	C15—C14—H14	120.4
C4—C5—H5	120.0	C14—C15—C11	119.02 (14)
C6—C5—H5	120.0	C14—C15—H15	120.5
C7—C6—C5	119.49 (13)	C11—C15—H15	120.5
C7—C6—H6	120.3	C12—C16—H16A	109.5
C5—C6—H6	120.3	C12—C16—H16B	109.5
C6—C7—C8	120.32 (13)	H16A—C16—H16B	109.5
C6—C7—H7	119.8	C12—C16—H16C	109.5
C8—C7—H7	119.8	H16A—C16—H16C	109.5
C3—C8—C7	120.53 (13)	H16B—C16—H16C	109.5
C3—C8—H8	119.7		
			
C1—N1—N2—C9	−177.73 (12)	N1—N2—C9—C11	−1.3 (2)
N2—N1—C1—S1	179.30 (10)	N1—N2—C9—C10	179.55 (11)
N2—N1—C1—S2	−1.64 (16)	C12—N3—C11—C15	−0.14 (19)
C2—S2—C1—N1	−175.99 (10)	C12—N3—C11—C9	−178.65 (12)
C2—S2—C1—S1	3.02 (11)	N2—C9—C11—N3	−6.2 (2)
C1—S2—C2—C3	171.62 (9)	C10—C9—C11—N3	172.96 (12)
S2—C2—C3—C8	−107.95 (12)	N2—C9—C11—C15	175.28 (14)
S2—C2—C3—C4	68.72 (14)	C10—C9—C11—C15	−5.57 (19)
C8—C3—C4—C5	−0.2 (2)	C11—N3—C12—C13	0.81 (19)
C2—C3—C4—C5	−176.92 (12)	C11—N3—C12—C16	−179.56 (12)
C3—C4—C5—C6	0.0 (2)	N3—C12—C13—C14	−0.7 (2)
C4—C5—C6—C7	−0.1 (2)	C16—C12—C13—C14	179.73 (13)
C5—C6—C7—C8	0.3 (2)	C12—C13—C14—C15	−0.2 (2)
C4—C3—C8—C7	0.4 (2)	C13—C14—C15—C11	0.8 (2)
C2—C3—C8—C7	177.14 (12)	N3—C11—C15—C14	−0.7 (2)
C6—C7—C8—C3	−0.4 (2)	C9—C11—C15—C14	177.80 (13)

### Hydrogen-bond geometry (Å, º)

Cg1 and Cg2 are the centroids of the (N3,C11–C15) and (C3—C8) rings, respectively.

**Table:**

*D*—H···*A*	*D*—H	H···*A*	*D*···*A*	*D*—H···*A*
N1—H1*N*···N3	0.87 (1)	1.91 (2)	2.6123 (16)	138 (1)
C8—H8···S2^i^	0.95	2.90	3.6184 (16)	154
C2—H2*A*···*Cg*1^ii^	0.99	2.76	3.5325 (15)	135
C7—H7···*Cg*2^iii^	0.95	2.89	3.5760 (16)	130
C10—H10*C*···*Cg*2^iv^	0.98	2.68	3.5842 (15)	153

Symmetry codes: (i) *x*, *y*−1, *z*; (ii) *x*, −*y*−1/2, *z*−3/2; (iii) −*x*, *y*−1/2, −*z*+1/2; (iv) *x*, −*y*−1/2, *z*−1/2.
